# COVID-19 Vaccine Intention among Healthcare Workers in Saudi Arabia: A Cross-Sectional Survey

**DOI:** 10.3390/vaccines9080835

**Published:** 2021-07-29

**Authors:** Mohammed Noushad, Mohammad Zakaria Nassani, Anas B. Alsalhani, Pradeep Koppolu, Fayez Hussain Niazi, Abdulaziz Samran, Samer Rastam, Ali Alqerban, Ali Barakat, Hesham S. Almoallim

**Affiliations:** 1 Department of Restorative and Prosthetic Dental Sciences, College of Dentistry, Dar Al Uloom University, Riyadh 11512, Saudi Arabia; mznassani@dau.edu.sa (M.Z.N.); fayez.h@dau.edu.sa (F.H.N.); asamran@dau.edu.sa (A.S.); ali.ab@dau.edu.sa (A.B.); 2 Biomaterials Unit, School of Dental Sciences, Health Campus, Universiti Sains Malaysia, Kubang Kerian 16150, Malaysia; 3 Department of Oral Medicine and Diagnostic Sciences, Vision College of Dentistry and Nursing, Vision Colleges, Riyadh 11691, Saudi Arabia; Dr.anas_salhany@hotmail.com; 4 Department of Preventive Dental Sciences, College of Dentistry, Dar Al Uloom University, Riyadh 13313, Saudi Arabia; pradeep@dau.edu.sa (P.K.); a.alqerban@psau.edu.sa (A.A.); 5 Department of Prosthodontics, College of Dentistry, Ibb University, Ibb 70270, Yemen; 6 Department of Biomedical Sciences, Vision College of Medicine, Vision Colleges, Riyadh 11691, Saudi Arabia; mrastam@gmail.com; 7 Department of Preventive Dental Sciences, College of Dentistry, Prince Sattam Bin Abdulaziz University, Al-Kharj 11942, Saudi Arabia; 8 Department of Surgical and Diagnostic Sciences, College of Dentistry, Dar Al Uloom University, Riyadh 13313, Saudi Arabia; halmoallim@dau.edu.sa; 9 Department of Oral and Maxillofacial Surgery, College of Dentistry, King Saud University, Riyadh 11545, Saudi Arabia

**Keywords:** COVID-19, vaccine intention, herd immunity, Saudi Arabia, healthcare workers

## Abstract

The COVID-19 pandemic has caused largescale morbidity and mortality and a tremendous burden on the healthcare system. Healthcare workers (HCWs) require adequate protection to avoid onward transmission and minimize burden on the healthcare system. Moreover, HCWs can also influence the general public into accepting the COVID-19 vaccine. Therefore, determining COVID-19 vaccine intention among HCWs is of paramount importance to plan tailor-made public health strategies to maximize vaccine coverage. A structured questionnaire was administered in February and March 2021 among HCWs in Saudi Arabia using convenience sampling, proceeding the launch of the vaccination campaign. HCWs from all administrative regions of Saudi Arabia were included in the study. In total, 674 out of 1124 HCWs responded and completed the survey (response rate 59.9%). About 65 percent of the HCWs intended to get vaccinated. The intention to vaccinate was significantly higher among HCWs 50 years of age or older, Saudi nationals and those who followed the updates about COVID-19 vaccines (*p* < 0.05). The high percentage (26 percent) of those who were undecided in getting vaccinated is a positive sign. As the vaccination campaign gathers pace, the attitude is expected to change over time. Emphasis should be on planning healthcare strategies to convince the undecided HCWs into accepting the vaccine in order to achieve the coverage required to achieve herd immunity.

## 1. Introduction

The SARS-CoV-2 virus outbreak that causes coronavirus disease-19 (COVID-19) is one of the largest pandemics to affect humankind during the last century. Governments scrambled to impose strict preventive measures such as complete, partial or phased lockdowns, social distancing, use of face masks, etc. Although these measures have been proven to slow down the transmission of the disease, it does lead to indirect mortality and morbidity due to other diseases, and a huge socio-economic burden on the population [1]. Therefore, the ideal path to the eradication of COVID-19 is through mass vaccination campaigns.

Even though vaccines have been proven to help control the spread and reduce the mortality rates of infectious diseases and are safer and more effective than therapeutics, there are indications of a decline in vaccine acceptance in many parts of the world [2,3]. Currently, several vaccines have been developed and approved by regulatory bodies and governments in a short time due to the urgency in dealing with the COVID-19 pandemic. For example, by mid-December 2020, the Pfizer-BioNTech vaccine (BNT162b2) with a reported efficacy of 95.0% was approved by regulatory bodies in several countries, while the Moderna vaccine (mRNA-1273) with a reported efficacy of 94.5% was approved for emergency use in the United States with several other nations following suit [4]. However, apart from factors, such as lack of access to vaccines, especially in low-income countries, rising anti-vaccination attitudes or vaccine hesitancy could prove to be a hindrance in achieving sufficient vaccine coverage to effectively limit the transmission of the virus. For example, a systematic review of vaccine acceptance among HCWs worldwide suggested acceptance rates ranging from 27.7% in the Democratic Republic of the Congo to 78.1% in Israel [5].

Vaccine hesitancy, a delay in vaccine acceptance or its refusal despite its availability, has been considered by the World Health Organization (WHO) as a global health threat in 2019 [6]. Contributing factors to vaccine hesitancy include, lack of confidence in and fear of vaccines, a negative perception on the need for vaccines (possibly due to disease severity underestimation), difficulties in accessing the vaccine, etc. [7]. Therefore, apart from the efficacy of the vaccine itself, the real success in combating the COVID-19 pandemic depends largely on not only implementation of well-planned mass vaccination campaigns but also on its large-scale acceptance by the population. Investigating the determinants of vaccination behavior is, therefore, important to plan effective vaccination strategies.

Since HCWs form the frontline combat force against COVID-19, and therefore fall in the high-risk group, several nations have included them in the high priority group as part of their first phase of vaccination programs [8]. Not only are they at increased risk of becoming infected themselves, but also of onward transmission to colleagues, hospital attendees, family members, etc. For example, a study in a tertiary care hospital in Saudi Arabia employing more than 16,000 HCWs indicated that they were ten times at higher risk of COVID-19 infection than non-HCWs [9]. Moreover, infection of HCWs will cause a temporary shortage in the frontline workforce leading to inefficiencies in pandemic management and a possible collapse of the healthcare system in extreme conditions. Vaccine acceptance among HCWs will not only protect them from the disease but will also help in building public confidence in vaccines, as they are generally considered trustworthy sources for promoting credible information on the importance of vaccines [10]. Therefore, understanding the determinants that can predict the intention of HCWs’ to accept or decline the COVID-19 vaccine is of paramount importance for planning successful vaccination campaigns. The aim of this study was to evaluate the intention of the HCWs in Saudi Arabia to take the COVID-19 vaccine and to indicate the determinants that may influence vaccine hesitancy in them.

## 2. Materials and Methods

### 2.1. Study Design

A cross-sectional online self-administered survey was conducted among HCWs in Saudi Arabia in the months of February and March 2021. The ‘Report of the sage working group on vaccine hesitancy’ was used as a guide in preparing the questionnaire [11]. As part of validation of the study, a pilot study was initially carried out on 10 participants, after which expert opinion was taken from four specialists in the field. Forms completed as part of the pilot study were not included in the actual study. The survey questionnaire developed on Google Forms was distributed through WhatsApp. The questionnaire required less than five minutes to complete. Participation was voluntary and the participants provided informed consent on the survey platform before proceeding to the survey items. The participants’ anonymity was guaranteed during the data collection process. Participants were reminded once, on failure to complete the survey form.

This study was approved by the Research Committee of College of Dentistry, Dar Al Uloom University, Saudi Arabia (COD/IRB/2020/2).

### 2.2. Sample

Power of the sample size was calculated using Open Source Epidemiologic Statistics for Public Health–OpenEpi (http://www.openepi.com/Menu/OE_Menu.htm, accessed 15 May 2021). The current study used the “Sample Size for Proportion” module with 50% as anticipated frequency that is recommended for unknown frequency, and 5% absolute precision. The result was a sample size of 664 to get a 99% confidence interval, which was used in the current study.

Participants (n = 674) included HCWs from various specialties. HCWs expected to come in contact with patients were included in the study. Students who had entered the clinical training level in their fields of study were also included in the study. Due to the greater number of HCWs in the central region, majority of the participants were from this region. However, participants from all other administrative regions of Saudi Arabia were also included in the study. Participants below the age of 18 years were not included in the study. Participants were not paid compensation for participation in the study. The survey form was designed in such a way that only completed forms would qualify for submission.

### 2.3. Measures

#### 2.3.1. Trust in Vaccines, Vaccine Manufacturers, and Health Authorities

General attitudes toward vaccines were measured using a 6-item scale. First, participants were asked if vaccines were really necessary to overcome the pandemic. Assessment questions on perceived trust in vaccine manufacturers included, whether participants trusted vaccines of only specific companies, whether vaccine manufacturers followed recommended development and production guidelines, concerns of commercial profiteering, risk of side effects of vaccines and whether manufacturers were open about disclosing the side effects of vaccines. Responses were rated on a five-point scale from 1 “strongly agree” to 5 “strongly disagree”. Participants’ attitudes toward health care system were measured using a 2-item scale. Participants were asked if they were happy with the health authorities’ handling of the pandemic, and their management of vaccination campaigns. Responses were rated on a five-point scale from 1 “strongly agree” to 5 “strongly disagree”.

#### 2.3.2. Intention to Vaccinate

The vaccine intention was measured using a 7-item scale. First, participants were asked if vaccines should be made mandatory and if the participant intended to get vaccinated. Further questions included fear of the vaccine, care for others who would be in greater need for the vaccine, intention to protect others with weaker immunity, willingness/unwillingness to take the vaccine if required to pay for it, and fear of side effects from second dose. Responses were rated on a six-point scale from 1 “strongly agree” to 5 “strongly disagree”.

### 2.4. Exploratory Variables

Socio-demographic factors included age group, sex, nationality, region of current work/study place, type of work/study (governmental/private/both), and profession. Participants’ report on chronic medical condition (e.g., asthma, diabetes, hypertension, heart disease, and/or cancer) was used to indicate the presence or absence of pre-existing co-morbidity. Other variables included, participants’ self-updating on COVID-19 vaccine development, prior infection with COVID-19, perception of COVID-19 severity, compliance with government COVID-19 guidelines and anxiety toward contracting COVID-19.

### 2.5. Statistical Analysis

Descriptive univariate analyses were conducted and were expressed as percentages and numbers for each item/survey question. The main outcome of this study was intention to vaccinate. The current study considered any participant to have an intention to vaccinate if he/she agreed or strongly agreed on the item “I will get vaccinated with the COVID-19 vaccine”, or if they have already taken the vaccine. Bivariate statistical analysis of the relationship between the main outcome “intention to vaccinate” and factors was done using the Chi-squared test for trend for ordinal factors, and Chi-squared test for categorical variables. A multivariate binary logistic regression model was used to determine the predictors for intention to vaccinate. The following factors were examined as potential predictors for the intention to vaccinate: age group, sex, nationality, presence of any medical condition, previous infection with COVID-19, following updates on the development of vaccines against COVID-19, opinion about the severity of COVID-19, compliance with COVID-19 preventive guidelines, and anxiety about contracting COVID-19. All statistical analyses were performed using IBM SPSS Statistics version 25.0 (IBM Corp. Released 2017. IBM SPSS Statistics for Windows, Version 25.0. IBM Corp, Armonk, NY, USA). The significance level was set at *p* < 0.05.

## 3. Results

The sociodemographic and working nature of the study sample is presented in Table 1. In total, 674 out of 1124 HCWs responded and completed the survey (response rate 59.9%). Table 2 shows that 15% of the respondents were previously infected with COVID-19. Of the participants, only 10% were vaccinated against COVID-19 at the time of the survey. As well, a high proportion of participating HCWs have been updating themselves about the development of COVID-19 vaccines (83.5%). In terms of severity of COVID-19, almost half of the sample rated it as severe disease (49.6%) and 70% expressed their compliance with COVID-19 preventive guidelines. Approximately a fifth of the recruited HCWs indicated high anxiety level about contracting COVID-19 (22.1%) (Table 2).

While a clear majority of the study population agreed that vaccines against COVID-19 are important to overcome the pandemic and bring life back to normal (73.3%), trust in the available vaccines was less than optimal with almost 43% of the participants indicating trust in the vaccines of only certain companies and 46% being not sure. Some doubts about the vaccines were also expressed by a considerable proportion of the respondents as 34% thought that the vaccines have been produced in a hurry without following recommended guidelines and 38% believed that money was the motive for companies to develop the vaccines. In addition, 45% indicated their fears of potential side effects for COVID-19 vaccines. On the level of trust in the Health Authorities overall management of COVID-19 pandemic, a positive attitude was predominant among study group. The above results are summarized in Table 3.

Support for a mandatory vaccination program against COVID-19 was shown by around 59% of the participants. Additionally, 64.1% indicated their intention to get vaccinated/have been vaccinated. On the contrary, 29% of the HCWs indicated their fear of taking the vaccine. Awareness regarding the importance of taking the vaccine as a mean for protection of the patients was high (78%). While 31% of the surveyed HCWs indicated their intention to take the COVID-19 vaccine only if it is free, 37% indicated the intention to take it even if they had to pay for it. In addition, more than half of the participants expressed their readiness to delay taking the vaccine in favor of people who are in greater need for the vaccination (54.5%), and only 28% had some concerns about possible side effects from the second dose of the vaccine. Table 4 illustrates the abovementioned findings.

The bivariate statistical analysis indicated an association between participants’ intention to vaccinate and six factors (Table 5). It can be noted that the intention to vaccinate increased significantly with increasing age (*p* < 0.05). Additionally, male subjects showed significantly higher intention to vaccinate than females (*p* < 0.05). Furthermore, there has been an association between the intention to vaccinate and updating self on the development of COVID-19 vaccines/opinion about the severity of COVID-19 (*p* < 0.05). Greater compliance with COVID-19 preventive guidelines and higher level of anxiety about contracting COVID-19 were associated with greater intention to vaccinate (*p* < 0.05).

The logistic regression analysis indicated three predictors for the intention to vaccinate among HCWs in Saudi Arabia. These include age, nationality and updating self on the development of vaccines against COVID-19. The intention to vaccinate was significantly higher among HCWs 50 years of age or older, Saudi nationals and those are who followed the updates about COVID-19 vaccines (*p* < 0.05) (Table 6).

## 4. Discussion

Understanding vaccine hesitancy in Saudi Arabia is crucial as it is home to Islam’s holiest sites and attracts millions of pilgrims throughout the year from all over the world. It is reported that more than one-and-a-half million worshippers have visited the Grand Mosque in Makkah during the first 10 days of the holy month of Ramadhan alone [12]. The current study was therefore conducted to determine the intention of HCWs in Saudi Arabia to take the COVID-19 vaccine. In the current study, 65 per cent of the HCWs had either taken the vaccine or agreed to do so. However, 73 percent of the HCWs agreed that a vaccine was necessary to end the pandemic. On the positive side, only 9 percent disagreed to take the vaccine, the remaining 26 percent being undecided.

Saudi Arabia launched its COVID-19 vaccination campaign on 17 December 2020 with the Minister of Health taking the first shot as a sign of building confidence among the HCWs and the public [13]. Registration for the vaccination was made possible through the ‘Sahhaty’ and ‘Tawakkalna’ applications, and the decision was taken to roll out the vaccines for free. Although not mandatory, HCWs were encouraged to take the vaccine and it was made a prerequisite for those HCWs participating in the 2021 Hajj (Pilgrimage) Season [14].

The percentage of those willing to be vaccinated in this study is similar to a previous study conducted on the Saudi general population that showed that 64% of the respondents were willing to get vaccinated against COVID-19 [15]. The result is also in agreement with a study done on the general population, in the neighboring country of Kuwait where 67 percent of the respondents were willing to take the vaccine [16]. However, the intention was much higher than that of another study on HCWs in Saudi Arabia in which only about 50 percent of the respondents were willing to take the vaccine against COVID-19 [17]. Additionally, compared to another similar study on the general population in Saudi Arabia in which only about 48 percent of the participants intended to get vaccinated, the acceptance rate in the current study is significantly higher [18]. The discrepancy between the current and the previous two studies could be due to the variation in the timing of the studies. The previous studies were conducted just before the launch of the vaccination campaign and when the number of new infections were diminishing tremendously, while the current study was conducted after the launch and promotion of the vaccination campaigns. Moreover, during the study period, the Ministry of Health have been actively engaged in regularly updating the population on the vaccination campaigns through mobile messaging applications and through social and digital media which could have increased awareness during the study period.

The current study indicated certain demographic factors associated with higher intention to get vaccinated against COVID-19. These include being male, Saudi national and being older than 50 years. Several studies have suggested that being female HCW decreased the odds of agreeing to take the vaccine [19,20]. The same was shown to be true in non-HCWs as well [21]. A pervious study on HCWs in Saudi Arabia indicated that males were more likely to accept the vaccine [18]. This trend was also confirmed by a report in the local newspaper which indicated that the turnout for vaccination among women was below the expected level even though 55 percent of the infections in Saudi Arabia were among women [22]. It could possibly be due to differences in risk perception between the sexes, the high mortality rate among males due to COVID-19, etc. [23].

The intention to get vaccinated was shown to increase with increasing age. This could be a result of an increased risk perception among the older age group, due to the fact that morbidity and mortality to COVID-19 increases with advancing age [24]. A positive relationship between HCWs as well as non-HCWs with increasing age and COVID-19 vaccine acceptance has been shown in other studies as well [20,21]. A study on the Saudi Arabian population indicated that individuals above 45 years of age were more likely to take the COVID-19 vaccine. Other studies on HCWs in France and Turkey also revealed that the intention to get vaccinated increased with increasing age, which is similar to the results of the current study [25].

The current study indicated a correlation between the perception of the severity of COVID-19 and the intention to vaccinate. The intention to vaccinate increased with increasing severity perception of the disease. A study on HCWs in China showed a similar correlation [26]. Other factors that have shown correlation with intention to vaccinate include compliance with COVID-19 preventive guidelines and anxiety of contracting the disease. A study in neighboring Kuwait concluded that people who were compliant with containment policies had greater odds of vaccine acceptance [16]. A large study in the UK also revealed similar results, in which poor compliance with COVID-19 guidelines was associated with negative vaccine views [27]. A previous study on HCWs in Saudi Arabia suggested that those who perceived a high or very high risk of infection with COVID-19 were more likely to accept the vaccine [17]. Similarly, a study on the general population of Saudi Arabia also reported that participants who were concerned about becoming infected were 2.13 times more likely to take the vaccine [15]. In conformity with our results, studies based on the Health Belief Model have suggested that being male, older in age, perceived severity of COVID-19, perceived susceptibility, compliance with preventive guidelines, etc., were associated with greater intention to vaccinate [28,29].

Multivariate logistic regression analysis was carried out to pinpoint possible predictors of vaccine hesitancy in HCWs in Saudi Arabia. Advancing age, being Saudi national and updating self on the development of vaccines against COVID-19 were significantly associated with the willingness to get vaccinated against COVID-19. In relation to nationality, a study on COVID-19 vaccine hesitancy in the general public in Saudi Arabia showed that a greater number of non-Saudi’s expressed their intention to take the COVID-19 vaccine than Saudis [15]. The same was true with seasonal influenza vaccine uptake in the general population in Saudi Arabia [30]. These results are contradictory to the results of the current study, which could be due to the variation in the study population. In an exploratory descriptive study including HCWs in India in which the doctors updated their knowledge of COVID-19 vaccines based on current research and government policies, they were shown to have a positive outlook toward vaccines. Similar results were achieved for other hospital staff who did it based on information from doctors and paramedics [31]. Although vaccines against COVID-19 were developed in a short period of time, their high efficacy has been proven by several studies. This could be a reason HCWs in the current study who followed the development of vaccines were more likely to accept the vaccine.

Although only 65 percent of the HCWs in the current study were willing to take the vaccine, the high percentage of the undecided ones (26 percent) is a positive sign as it would be easier to convince them to take the vaccine than the ones who disagree. The higher rate of infection and greater vaccine hesitancy among women, and the nationality dependent vaccine intention, is something that needs to be studied further and tackled accordingly. The high level of trust in the health authorities in managing the vaccination campaign and the pandemic in general is another positive sign. The tremendous efforts of the authorities is evident from the fact that as of 23 June 2021, Saudi Arabia has vaccinated more than 70 percent of its adult population and begun vaccinating individuals in the 12–18 years age group [32]. Since HCWs are considered credible sources of health information and are held in high confidence by the general public, policymakers must initiate efficient strategies to minimize vaccine misinformation among them. They can then act as a link between policymakers and the general public in dissemination and communication of positive messages on the COVID-19 vaccine and its importance in attaining population immunity.

The current study has a few strengths. It is the first study on HCWs’ intention to vaccinate against COVID-19, after the launch of the vaccination campaign. The participants in the current study comprised of a wide distribution of HCWs from various specialties. The study also covered all major regions of Saudi Arabia.

The current study has a few limitations as well. Since the participants were recruited using convenience sampling the representativeness of the samples is relatively low. The web-based self-administration of the survey could result in potential bias among the participants in responding to the survey questions. However, due to the restrictions related to the pandemic, this was the best mode currently available. Since the study was carried out in the beginning of the vaccination campaign, the intention could change considerably over time. Therefore, further studies will provide added value on the evolving vaccine intention trend among HCWs in Saudi Arabia.

## Figures and Tables

**Table 1:** Characteristics of participants (No = 674).

Parameter	Category	N (%)
**Age (years)**	18–29 y	392 (58.2)
	30–49 y	251 (37.2)
	50–64 y	27 (4)
	Above 64 y	4 (0.6)
**Sex**	Male	350 (51.9)
	Female	324 (48.1)
**Nationality**	Saudi	410 (60.8)
	Non-Saudi	264 (39.2)
**Current work/study place in Saudi Arabia**	Northern Region	14 (2.1)
	Southern Region	10 (1.5)
	Central Region	555 (82.3)
	Eastern Region	60 (8.9)
	Western Region	35 (5.2)
**Type of Practice**	Governmental	154 (22.8)
	Private	488 (72.4)
	Both	32 (4.7)
**Medical Condition**	Healthy	573 (85)
	Has chronic disease/s	101 (15)
**Profession**	Medical doctor	76 (11.3)
	Dentist	191 (28.3)
	Nurse	41 (6.1)
	Pharmacist	29 (4.3)
	Dental assistant/Hygienist Medical	6 (0.9)
	/Dental technician	6 (0.9)
	Health care student	238 (35.3)
	Other health profession	87 (12.9)

**Table 2:** General questions regarding COVID-19.

No	Question	Participants’ Response *n (%)*
**1**	Have you had COVID-19?	Yes 102 (15.1) No 572 (84.9)
**2**	Have you taken the COVID-19 vaccine?	Yes 67 (9.9) No 607 (90.1)
**3**	Have you been updating yourself on the development of vaccines against COVID-19?	Yes 563 (83.5) No 111 (16.5)
**4**	In your opinion, how would you rate the severity of COVID-19?	Mild 25 (3.7) Moderate 315 (46.7) Severe 334 (49.6)
**5**	How would you rate your compliance with COVID-19 preventive guidelines?	Good 472 (70) Moderate 188 (27.9) Poor 14 (2.1)
**6**	To what extent are you anxious about contracting (getting infected with) COVID-19?	High 149 (22.1) Moderate 364 (54) Low 161 (23.9)

**Table 3:** Trust in COVID-19 Vaccines and Health Authorities.

No	Statement	Participants’ Response *n (%)*				
		Strongly Agree	Agree	Not Sure	Disagree	Strongly Disagree
**1**	Vaccines are necessary to overcome the COVID-19 pandemic and get back to normal life.	283 (42)	211 (31.3)	148 (22)	17 (2.5)	15 (2.2)
**2**	I trust COVID-19 vaccines of ONLY certain companies.	129 (19.2)	161 (23.9)	310 (46)	40 (5.9)	34 (5)
**3**	I think that vaccines against COVID-19 have been produced in a hurry without following recommended clinical trials and approval guidelines.	81 (12)	149 (22.1)	247 (36.7)	124 (18.4)	73 (10.8)
**4**	I think that the companies involved in the development of the COVID-19 vaccines are doing it to make money.	85 (12.6)	172 (25.5)	273 (40.5)	107 (15.9)	37 (5.5)
**5**	I think that COVID-19 vaccines may have side effects which may show immediately or later on in life.	82 (12.1)	220 (32.6)	304 (45.1)	56 (8.3)	12 (1.8)
**6**	I think companies producing COVID-19 vaccines are open about disclosing information on the side effects of the vaccine.	62 (9.2)	212 (31.5)	325 (48.2)	61 (9.1)	14 (2)
**7**	I am happy with the way the health authorities have been managing the COVID-19 pandemic so far.	409 (60.7)	211 (31.3)	35 (5.2)	14 (2.1)	5 (0.7)
**8**	I am happy with the health authorities’ efficient organization of the COVID-19 vaccination campaigns through the digital applications and other methods.	378 (56.1)	240 (35.6)	43 (6.4)	8 (1.2)	5 (0.7)

**Table 4:** Intention to vaccinate.

No	Statement	Participants’ Response *n (%)*				
		Strongly Agree	Agree	Not Sure	Disagree	Strongly Disagree
**1**	I support a mandatory vaccination program for COVID-19.	227 (33.6)	169 (25.1)	152 (22.6)	80 (11.9)	46 (6.8)
**2**	I will get vaccinated with the COVID-19 vaccine.	273 (40.5)	159 (23.6)	180 (26.7)	40 (5.9)	22 (3.3)
**3**	I will wait for other people to take the COVID-19 vaccine, as I am afraid to take it myself.	55 (8.2)	139 (20.6)	147 (21.8)	193 (28.6)	95 (14.1)
**4**	I will delay taking the COVID-19 vaccine, as I feel there are others who deserve it more than me.	124 (18.4)	243 (36.1)	135 (20)	82 (12.2)	45 (6.7)
**5**	Getting myself vaccinated against COVID-19 is important because I can also protect people with a weaker immune system.	271 (40.2)	255 (37.8)	115 (17.1)	17 (2.5)	14 (2.1)
**6**	I will take the COVID-19 vaccine only if it is free.	97 (14.4)	113 (16.8)	169 (25.1)	163 (24.2)	85 (12.6)
**7**	Compared to the first dose of the COVID-19 vaccine, I fear that the second dose may have more chances to induce adverse side effects.	54 (8)	136 (20.2)	320 (47.5)	93 (13.8)	33 (4.9)

**Table 5:** Bivariate statistical analysis of the relationship between the main outcome “intention to vaccinate” and potential influential factors.

	*% (n)*	*p*
**All Participants**	64.4% (433/672)	
**Age**		0.015 *
18–29 years	61.5% (241/392)	
30–49 years	66.7% (166/249)	
50 years or above	83.9% (26/31)	
**Sex**		0.034 **
Male	68.2% (238/349)	
Female	60.4% (195/323)	
**Nationality**		0.08 **
Saudi	67% (274/409)	
Non-Saudi	60.5% (159/263)	
**Medical condition**		0.30 **
Healthy	63.6% (364/572)	
Has systemic disease/s	69% (69/100)	
**Previously infected with COVID-19**		0.08 **
No	65.8% (375/570)	
Yes	56.9% (58/102)	
**Updating self on the development of vaccines against COVID-19**		<0.001 **
No	49.5% (55/111)	
Yes	67.4% (378/561)	
**Opinion about the severity of COVID-19**		0.001 *
Mild	48% (12/25)	
Moderate	59.4% (186/313)	
Severe	70.4% (235/334)	
**Compliance with COVID-19 preventive guidelines**		0.018 *
Poor	42.9% (6/14)	
Moderate	59.7% (111/186)	
Good	66.9% (316/472)	
**Anxiety about contracting COVID-19**		0.011 *
Low	55.3% (89/161)	
Moderate	66.7% (242/363)	
High	68.9% (102/148)	

*: *p* was calculated using chi-square test for trend. **: *p* was calculated using chi-square test. Significance difference was set at *p* < 0.05.

**Table 6:** Predictors of intention to vaccinate.

	Odds Ratio (95% Confidence Interval)	*p*
**Age**		
18–29 years	Ref	
30–49 years	1.31 (0.87–1.96)	0.20
50 years or above	2.86 (1.01–8.06)	0.047 *
**Sex**		
Male	Ref	
Female	0.72 (0.51–1.01)	0.054
**Nationality**		
Saudi	Ref	
Non-Saudi	0.58 (0.40–0.85)	0.005 *
**Medical condition**		
Healthy	Ref	
Has systemic disease/s	1.09 (0.67–1.78)	0.73
**Previously infected with COVID-19**		
No	Ref	
Yes	0.76 (0.48–1.19)	0.23
**Updating self on the development of vaccines against COVID-19**		
No	Ref	
Yes	1.90 (1.23–2.94)	0.004 *
**Opinion about the severity of COVID-19**		
Mild	Ref	
Moderate	1.41 (0.59–3.36)	0.44
Severe	1.86 (0.76–4.55)	0.17
**Compliance with COVID-19 preventive guidelines**		
Poor	Ref	
Moderate	2.02 (0.65–6.28)	0.22
Good	1.74 (0.55–5.48)	0.35
**Anxiety about contracting COVID-19**		
Low	Ref	
Moderate	1.40 (0.84–2.32)	0.19
High	1.42 (0.94–2.13)	0.09

Odds ratio and 95% confidence interval was calculated by a binary logistic model. * Significant difference at *p* < 0.05.

## Data Availability

The questionnaire used in the current study is not publicly available due to certain restrictions. However, it is available from the corresponding author (Mohammed Noushad) on reasonable request.
